# Musculoskeletal, Pulmonary, and Cardiovascular COVID-19 Sequelae in the Context of Firefighter Occupational Health: A Narrative Review

**DOI:** 10.3390/ijerph21101383

**Published:** 2024-10-19

**Authors:** Elliot L. Graham, Susanne D’Isabel, Adriana Lofrano-Porto, Denise L. Smith

**Affiliations:** 1 Integrative Cardiovascular Physiology Laboratory, Colorado State University, Fort Collins, CO 80526, USA; 2 Intestinal Health Laboratory, Colorado State University, Fort Collins, CO 80526, USA; 3 First Responder Health and Safety Laboratory, Department of Health and Human Physiological Sciences, Skidmore College, Saratoga Springs, NY 12866, USA; sdisabel@skidmore.edu; 4 Molecular Pharmacology Laboratory, Health Sciences School, University of Brasilia, Brasilia 70910-900, DF, Brazil; 5 Endocrine Diseases Clinics, University Hospital of Brasilia, Brasilia 70840-901, DF, Brazil

**Keywords:** SARS-CoV-2, post-COVID-19 sequelae, long COVID, post COVID conditions, firefighting, fire service, emergency medical services, return to work, occupational readiness, cardiorespiratory fitness

## Abstract

For most individuals infected with SARS-CoV-2, the acute illness resolves completely. However, for millions of people, symptoms or sequelae from COVID-19 recur or persist for months to years after infection. Post-COVID-19 sequelae are wide-ranging, often affecting the musculoskeletal, pulmonary, and cardiovascular systems. All who experience post-COVID-19 sequelae face significant challenges navigating home and work life. Occupations such as firefighting, however, are of particular concern given the strenuous nature of a job that relies on a healthy musculoskeletal, pulmonary, and cardiovascular system. Research has documented significant musculoskeletal impairment (including muscle weakness, pain, and fatigue), respiratory dysfunction (including reduced lung function, interstitial disease, and diffusion abnormalities), cardiovascular conditions (including cardiac events, ischemic disease, dysrhythmias, and infectious diseases), and diminished cardiorespiratory fitness that continues for months to years in some individuals. These persistent post-COVID-19 conditions may affect a firefighter’s ability to return to work, function at full capacity while at work, and potentially compromise firefighter health and public safety. This review, therefore, explores musculoskeletal, pulmonary, and cardiovascular sequelae post-COVID-19 and the impact of these sequelae on firefighter health and occupational readiness.

## 1. Background

COVID-19, a viral illness resulting from SARS-CoV-2 infection, can cause multiorgan damage during the acute stage of infection and can also result in long-term sequelae. The COVID-19 pandemic wreaked havoc around the world—causing more than seven million premature deaths worldwide [1], over one million in the United States alone [2], and widespread, sometimes severe, illness. Fortunately, current SARS-CoV-2 variants appear less lethal, and vaccines and therapeutics are now available. Despite this somewhat encouraging scenario, and a widespread desire to move past the COVID-19 pandemic, there are still millions of people around the world who are facing post-COVID-19 sequelae [3].

Post-COVID-19 conditions, or long COVID, are general terms to describe the persistence or recurrence of symptoms after the acute illness has resolved. However, post-COVID-19 conditions are not a distinct entity. Rather, different phenotypes/clusterings of ongoing conditions occur in many individuals, with some individuals struggling with muscular impairment and fatigue, some reporting persistence of respiratory issues, and some experiencing new or exacerbated cardiovascular conditions. Despite a substantial amount of research attempting to understand the sequelae of COVID-19, there is still considerable uncertainty about the long-term effects of COVID-19 illness on physiological function and health. In addition, synthesizing current research on post-COVID-19 sequelae is challenging because sequelae of COVID-19 can present as different phenotypes, vary over time, are influenced by the severity of initial illness, and are affected by other comorbidities and possibly by genetic factors [4].

Post-COVID-19 sequelae can affect anyone who has been infected by the SARS-CoV-2 virus, and can impact both work and home life. The potential impact on individuals returning to work is multifaceted and may be specific to work environment and responsibilities. Firefighters are an important occupational cohort to consider, as a decrease in their operational effectiveness (i.e., due to post-COVID-19 sequelae) may negatively affect public safety [5]. Although there are other occupational cohorts who also experience emotionally stressful—even traumatic—events and work extended and unpredictable shifts, firefighters are unique in that they perform strenuous muscular work and are exposed to environmental extremes. Indeed, research has clearly demonstrated that firefighting leads to high levels of cardiovascular and thermal strain, requires high levels of muscular work and energy expenditure, disrupts immune and endocrine function, and leads to a pro-coagulatory state [6,7,8,9]. Furthermore, firefighters have a high prevalence of obesity and hypertension, which may increase the risk of post-COVID-19 sequelae [10,11]. Thus, firefighters are a particular cohort that could develop severe post-COVID-19 sequelae. Not only do sequelae from COVID-19 threaten to affect the health of millions of firefighters around the world, but sequelae may interfere with their ability to function effectively if they do return to work.

Therefore, in this narrative review, we evaluate evidence on the effect of post-COVID-19 sequelae on the muscular, pulmonary, and cardiovascular systems. With this evidence, in addition to the literature that has directly investigated the effects of post-COVID-19 sequelae on firefighters, we consider the potential impact of post-COVID-19 sequelae on the health and operational readiness of firefighters.

## 2. Review Process

Our initial search of Google Scholar and PubMed, using key words, yielded little data regarding the impact of musculoskeletal, pulmonary, and cardiovascular COVID-19 sequelae on firefighter occupational health. Therefore, we broadened our search to include articles (original research and reviews) that examined the impact of COVID-19 on long-term musculoskeletal, pulmonary, and cardiovascular health, as these are key organ systems that are known to be fundamental in the performance of occupational tasks. We synthesized this information with the results of a search for articles that examined the effects of firefighting on the musculoskeletal, pulmonary, and cardiovascular systems. No date restrictions were placed on searches.

## 3. Firefighting as an Occupation of Specific Concern for Those with Post-COVID-19 Sequelae

Firefighting involves performing strenuous muscular work to perform critical tasks, such as advancing a charged hoseline, making entry into buildings through locked doors or roofs, and rescuing victims. The work is often performed in dangerous environments and can be accompanied by high levels of sympathetic nervous system activation. If firefighters are unable to accomplish their tasks in a timely manner, a fire may grow and result in greater risk of burn injury, asphyxiation, or building collapse. Firefighters face multiple hazards as they perform their work, including working in immediately dangerous to life and health (IDLH) environments, which require the use of a self-contained breathing apparatus (SCBA). Despite all the hazards that firefighters face, cardiovascular events (sudden cardiac events and stroke) are the leading cause of line-of-duty death, accounting for approximately 50% of line-of-duty deaths in most years [12]. In order for firefighters to safely and effectively perform their work, they must have healthy muscular, pulmonary, and cardiovascular systems.

**Figure 1:**
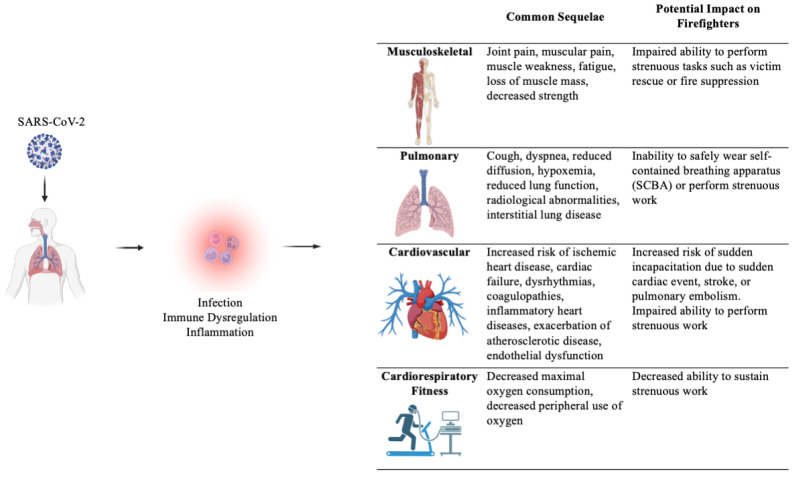
Long-Term Musculoskeletal Pulmonary, and Cardiovascular Sequelae of COVID-19 and Their Implications for Firefighter Health and Performance. Created with BioRender.com.

Acute SARS-CoV-2 infection can critically impair the muscular, pulmonary, and cardiovascular systems, and emerging evidence shows that there can be long-term sequelae affecting these systems. While post-COVID-19 sequelae can impair the functional ability of all workers, the consequences for firefighters are of particular concern for several reasons, including the following: firefighters must be able to endure strenuous work in order to perform their public safety mission; firefighters must be able to work while wearing a SCBA; the strenuous work of firefighting may trigger a sudden cardiac event in individuals with underlying disease; and emergency services are facing severe personnel issues due to recruitment and retention issues [13], which often results in mandatory overtime and is exacerbated by an increase in call volume. Figure 1 summarizes the musculoskeletal, pulmonary, and cardiovascular sequelae of COVID-19 and their potential impact on firefighters.

## 4. The Musculoskeletal System

Firefighting requires adequate strength and muscular function. Musculoskeletal impairments are a common sequela of COVID-19, and evidence indicates that the musculoskeletal system can be impaired long after the initial SARS-CoV-2 infection has subsided. In a small cohort study, a large percentage of COVID-19 patients had clinical musculoskeletal dysfunction at 3 months post-infection, including muscle weakness (50%), myopathic electromyography (75%), muscle fiber atrophy (38%), and skeletal muscle immune cell infiltration (62%) [14]. These findings are consistent with other studies that found reductions in lower- and upper-body strength, fatigue, reduced exercise tolerance, impaired muscle metabolism, and skeletal muscle mitochondrial dysfunction in those recovering from acute COVID-19 or with post-COVID-19 sequelae [15,16,17,18,19,20]. Stoffels et al. found evidence of muscle weakness at 14 weeks post-acute COVID-19 in approximately 60% of patients who were hospitalized or had post-acute sequelae of COVID-19 following mild illness [21]. Further, at 1.5 years, the prevalence of muscle weakness remained elevated in both groups. Musculoskeletal pain is also a common finding. In a comprehensive meta-analysis, four studies found that arthromyalgia (muscular and joint pain) was a common post-COVID-19 sequela with 26% (95% CI: 8 to 44) of subjects reporting it [22,23,24,25,26]. Pooled prevalence from five studies [20,27,28,29,30] in the same meta-analysis found 10% of subjects reported joint pain, 8% reported myalgia (eight studies) [20,27,28,29,30,31,32,33], and 4% reported backache/waist pain (three studies) [30,32,34]. Collectively, these studies provide strong evidence that musculoskeletal dysfunction, including decreased strength and increased muscle and joint pain, can persist for months to years following SARS-CoV-2 infection.

Researchers have sought to better understand who is at greater risk for persistent post-COVID-19 musculoskeletal impairments. It is well-documented that those with poor metabolic health are at an increased risk [35,36,37,38,39,40,41,42]. And, it is becoming clearer that persistent musculoskeletal impairment is also affected by the severity of acute COVID-19. For instance, Tanriverdi et al. compared upper- and lower-body weakness in those who had mild versus moderate COVID-19 [43]. At 12 weeks of recovery, those who had mild COVID-19 had significantly higher handgrip and quadricep strength than those who had moderate COVID-19 (dominant hand grip strength: 36.7 ± 12.9 kg vs. 28.2 ± 13.7 kg, *p* = 0.032; dominant quadricep strength: 27.9 ± 6.5 vs. 22.9 ± 7.3 kg, *p* = 0.03) [43]. As discussed above, Stoffels et al. studied outcomes in hospitalized and non-hospitalized patients and found sustained muscle weakness in both groups roughly 1.5 years post-COVID-19 [21]. In a study investigating sequelae for up to 2 years post-COVID-19, Bowe et al. found that musculoskeletal symptoms were still elevated 2 years after infection compared to non-infected controls [44]. Furthermore, although these sequelae impacted quality of life among both groups, the impact was more pronounced in the hospitalized cohort: 6.2 disability adjusted life years (DALYs) per 1000 persons (95% CI: 4.7–7.7) versus 3.2 DALYs per 1000 persons (95% CI: 2.8–3.6) among the non-hospitalized [44]. Thus, these data indicate that persistent musculoskeletal impairments following COVID-19 are affected by initial infection severity.

The magnitude of muscle loss and other musculoskeletal impairments during COVID-19 may reflect overall infection severity. Therefore, the degree of musculoskeletal dysfunction seen during COVID-19 could indicate the risk of persistent musculoskeletal issues following infection. Indeed, researchers have reported that greater muscle loss during COVID-19 hospitalization is indicative of greater prevalence of fatigue, myalgia, and muscle mass loss 6 months after hospital discharge compared to those with lower muscle loss during hospitalization (fatigue: 76% vs. 46%, *p* = 0.03; myalgia: 66% vs. 35%, *p* = 0.04, muscle mass: −8% vs. 3%, *p* < 0.01) [45]. This same study also showed that higher muscle loss was associated with greater total COVID-19-related healthcare costs 2 and 6 months after discharge. Researchers have also found that the myalgia experienced during the first week of SARS-CoV-2 infection was a significant predictor of the persistence of common COVID-19 symptoms (i.e., cough, abdominal pain, shortness of breath, fatigue) for more than 4 weeks. (OR 2.22, 95% CI: 1.80–2.73) [38].

The mechanism(s) underlying post-COVID-19 skeletal muscle dysfunction are incompletely understood, but it seems likely that several mechanisms could be acting in tandem. A heightened systemic immune response towards SARS-CoV-2 and direct infection of skeletal muscle via ACE2 and/or TMPSSR2 are both implicated in skeletal muscle dysfunction post-COVID-19 [46]. Furthermore, SARS-CoV-2 infection can impair skeletal muscle mitochondrial function that persists even after COVID-19 recovery. Recently, Appelman et al. [17] analyzed skeletal muscle biopsies in individuals with post-exertional malaise in the context of post-COVID-19 sequelae compared with fully recovered controls (no residual symptoms after SARS-CoV-2 infection). They found that compared to controls, patients with post-COVID-19 sequelae had similar reductions in oxidative phosphorylation capacity one day after exercise, but a significant decrease in succinate dehydrogenase (SDH) activity, and a metabolomic profile indicating a shift away from oxidative metabolism suggestive of impairments in mitochondrial respiration. A small cohort study found 62% of patients with post-COVID-19 symptoms persisting up to 14 months had reduced cytochrome c activity, abnormal mitochondrial structure, or larger subsarcolemmal mitochondrial accumulations in muscle biopsies [14]. In addition, Colosio et al. showed that long COVID patients had reduced mitochondrial respiratory oxygen flux for mitochondrial complex II compared to patients without long-term symptoms [16]. The post-COVID-19 group also had reduced mitochondrial ADP sensitivity and efficiency compared to the controls. Collectively, these studies provide strong evidence that SARS-CoV-2 infection can impair skeletal muscle mitochondrial oxygen utilization and energy production, which could be a key mechanism explaining muscular fatigue post-COVID-19.

Muscular strength and function are necessary for the occupational performance of firefighters. Firefighters have some of the highest incidences of musculoskeletal injuries among all Emergency Medical Service (EMS) personnel [47] with more than 60,000 injuries reported yearly—a number that is likely significantly underreported [48]. Post-COVID-19 musculoskeletal impairments may directly impact the ability of firefighters to perform occupational duties, increase their risk of musculoskeletal injuries, and potentially endanger public safety if firefighters are unable to complete critical tasks or do so in a timely manner.

## 5. The Pulmonary System

Respiratory dysfunction is a common sequela of COVID-19. Firefighters often work in IDLH environments and must, therefore, have adequate lung function and be able to wear positive pressure SCBAs. As the pulmonary system is particularly susceptible to SARS-CoV-2 infection, replication, inflammation, and injury, many of the lingering symptoms following SARS-CoV-2 infection are respiratory-related. Respiratory distress and/or pneumonia were among the most concerning clinical conditions of SARS-CoV-2 illness and accounted for a disproportionate number of deaths. Thus, it is not surprising that the majority of research related to the pulmonary system has focused on sequelae in individuals who had severe COVID-19, were hospitalized with COVID-19, or experienced prolonged symptoms. Research has found that hospitalized COVID-19 patients had persistent pulmonary dysfunction (i.e., reduced forced expiratory volume in 1 s (FEV1) and/or forced vital capacity (FVC)) after 6 weeks of recovery [49,50]. Other researchers have documented similar ailments including continuing pulmonary dysfunction, radiological abnormalities (including some evidence of fibrosis), and/or dyspnea 3–6 months post-infection [51,52,53,54,55]. In a large-scale study documenting sequelae among U.S. veterans who experienced COVID-19 compared to non-infected controls, it is apparent that even those who had a milder course of COVID-19 (were not hospitalized) have pulmonary sequelae of COVID-19 [44]. The occurrence of cough, dyspnea, hypoxemia and interstitial lung disease remained elevated at both 3 and 6 months post-infection. Thus, it is clear that the pulmonary sequelae of COVID-19 can persist for at least several months into COVID-19 recovery.

There is less clarity, however, on the long-term impact of SARS-CoV-2 on the pulmonary system. Some research has found improvements in pulmonary function and fewer aberrant radiological findings 6–12 months post-infection [50,54,56], while others have reported significant improvements in respiratory function and/or resolved pulmonary sequalae 1 year after initial illness [57]. It is important to note, however, that radiological abnormalities and pulmonary dysfunction were still prevalent even after 12 months in some of these studies [54,56]. For example, Eizaguirre et al. [54] showed that both the percent predicted mean total lung capacity and residual volume were lower at 12 months after hospital discharge from COVID-19 compared to 3 months (percent predicted mean total lung capacity at 3 months vs. 12 months: 97.2 vs. 92.9, *p* = 0.06; residual volume at 3 months vs. 12 months: 100.6 vs. 87.9, *p* < 0.01). This finding suggests a worsening of lung sequelae over time. In addition, 23.4% of patients still had abnormal FVC or Diffusing Lung Capacity for Carbon Monoxide (DLCO) findings after 12 months. These findings are consistent with data from Wu et al. [56], who found DLCO to be only 88% of the predicted (78% to 101%) 12 months after discharge from the hospital following COVID-19. Furthermore, 24% of the discharged patients still presented with abnormal radiological changes (high-resolution computed tomography (HRCT) after 12 months [56]. Interestingly, aberrant HRCT scores were significantly associated with increased length of hospital stay (*p* = 0.03) and peak HRCT pneumonia scores (*p* < 0.01). Significant differences in pulmonary function (DLCO, functional residual capacity, FVC, residual volume, total lung capacity, and vital capacity) were found between those with normal and abnormal HRCT scores at 12 months after discharge. These findings, in conjunction with sustained pulmonary dysfunction, radiological abnormalities, dyspnea, and/or increased risk of chronic pulmonary and interstitial lung disease as well as fibrosis found up to 1 year post-infection [58,59,60,61,62], suggest that pulmonary dysfunction can persist at least a year after moderate to severe COVID-19 illness.

The extent to which the severity of the initial SARS-CoV-2 infection predicts the severity or persistence of pulmonary sequelae is unclear. Several studies have found that pulmonary dysfunction and dyspnea were prevalent in both hospitalized and non-hospitalized patients at least 16 months post-infection [44,63,64,65]. Persistent pulmonary disorders were also shown to contribute to disease burden 2 years after infection in both hospitalized and non-hospitalized individuals (hospitalized: 44.9 DALYs per 1000 persons (95% CI: 41.3 to 48.8); non-hospitalized: 8.2 DALYs per 1000 persons (95% CI: 7.6 to 8.8)) [44]. These results suggest that pulmonary impairments are sustained long after COVID-19 infection in both hospitalized and non-hospitalized individuals. Most research indicates that initial infection severity does impact the severity of the pulmonary dysfunction seen after infection [53,63,66,67,68,69,70,71,72,73,74]. However, several studies have found no relationship between infection severity and pulmonary dysfunction [58,75,76]. Thus, more research is needed to understand the relationship between SARS-CoV-2 infection severity and post-COVID-19 pulmonary sequelae.

Pulmonary dysfunction and other comorbidities experienced before SARS-CoV-2 infection might influence the persistence of post-COVID-19 pulmonary impairments. Indeed, one review suggests that respiratory dysfunction prior to infection could exacerbate post-COVID-19 pulmonary dysfunction [77]. A retrospective study found that the number of pre-existing comorbidities was associated with post-COVID-19 dyspnea in hospitalized patients 2 years post-infection (OR 1.91, 95% CI: 1.04 to 3.58, *p* = 0.03) [78]. However, the lack of inclusion of uninfected controls limits the ability to assess the association of SARS-CoV-2 infection with symptoms 2 years after acute infection. It is, therefore, rational to presume that one’s overall health before infection is a potential determinant of post-infection pulmonary sequelae.

The pulmonary system is vital for the health and occupational performance of firefighters. Firefighters must have adequate lung function to carry out heavy work (including carrying an SCBA cylinder) while breathing air from their SCBA. Furthermore, firefighters have a risk of reduced respiratory health over their careers, including reduced spirometry values (FEV1 and FVC) as well as pulmonary fibrosis and interstitial lung disease [79,80,81,82], which could be the result of exposure to smoke and modern toxic combustion products [83,84,85,86,87,88,89]. Thus, firefighters are a cohort that could be in danger of developing exacerbated long-lasting pulmonary impairments if infected with SARS-CoV-2.

## 6. The Cardiovascular System

Although SARS-CoV-2 infection was initially viewed as a respiratory illness, it soon became apparent that SARS-CoV-2 infection had widespread detrimental effects on all components of the cardiovascular system, with discrete issues related to the heart, blood vessels, and blood. The effects of COVID-19 on the cardiovascular system are a particular concern for firefighters, as their occupational duties involve lengthy and strenuous work that places great demand on the heart and blood vessels and results in a procoagulatory state [8]. Multiple large-scale studies have shown a strong association between COVID-19 and acute cardiac events, dysrhythmias, heart failure, myocarditis, coagulopathy, microvascular injuries, arterial stiffness, and endothelial dysfunction [90,91,92], but these are not universal findings [91,93,94]. Individuals with preexisting cardiovascular complications or associates of cardiovascular diseases, such as aging, hypertension, obesity, and type 2 diabetes, are at an increased risk of developing cardiovascular sequelae [90].

It is well-established that cardiovascular risks are elevated in the immediate aftermath of acute COVID-19 illness. A large systematic review including over 8 million individuals (~1 million COVID-19 cases, ~7 million controls) from seven published studies evaluated the risk of a large number of cardiovascular abnormalities that are elevated following COVID-19, with the studies examining individuals from 1 month to 1 year after COVID-19. Major findings of this meta-analysis included higher pooled odds of myocarditis (OR 4.90, 95% CI, 3.55–6.24, *p* < 0.01), pulmonary embolism (OR 2.76, 95% CI: 2.50 to 3.02, *p* < 0.01), cardiac arrest (OR 2.08, 95% CI: 1.40 to 2.76, *p* < 0.01), atrial arrhythmia (OR 2.05, 95% CI: 1.24 to 2.85, *p* < 0.01), sinus tachycardia (OR 1.75, 95% CI: 1.60 to 1.91, *p* < 0.01), pericarditis (OR 1.72, 95% CI: 1.49 to 1.94, *p* < 0.01), ventricular arrhythmia (OR: 1.71, 95% CI: 1.48 to 1.95, *p* < 0.01), myocardial infarction (OR 1.60, 95% CI: 1.42 t0 1.78, *p* < 0.01) sinus bradycardia (OR 1.57, 95% CI: 1.50 to 1.63, *p* < 0.01), and stroke (OR 1.39, 95% CI: 1.15–1.63, *p* < 0.01) in those who had COVID-19 compared to non-infected controls [95].

Cohort studies have also investigated the persistence of cardiovascular sequelae months after infection. Tereshchenko et al. [96] looked at risk in the months following COVID-19 by conducting a retrospective double cohort study to compare individuals who tested positive for SARS-CoV-2 infection (symptomatic or asymptomatic) to those who were uninfected. Compared to the uninfected cohort, the SARS-CoV-2 positive cohort had a greater risk of the primary outcome (composite of cardiovascular morbidities and mortality) during a median of 6 months at risk (HR 1.71, 95% CI: 1.06 to 2.78, *p* = 0.03) [96]. Raisi-Estabragh et al. [97] investigated over 17,000 UK biobank cases between March 2020 and 2021, and showed that prior SARS-CoV-2 infection increased the risk of venous thromboembolism in both non-hospitalized (HR 2.74, 95% CI: 1.38 to 5.45, *p* < 0.01) and hospitalized individuals (HR 27.6, 95% CI: 14.5 to 52.3, *p* < 0.01) over an average of 141 days of follow-up after COVID-19. Hospitalized patients also had increased risks of heart failure (HR 21.6, 95% CI: 10.9 to 42.9, *p* < 0.01) and stroke (HR 17.5, 95% CI:5.26 to 57.9, *p* < 0.01).

Newer studies that have examined longer post-infection periods have shown that cardiovascular sequelae can persist even longer. Indeed, Xie et al. used a large U.S.-based cohort drawn from the Veterans Health Administration database and found that SARS-CoV-2 infection significantly increases 12-month risk of any cardiovascular outcome (HR 1.63; 95% CI 1.59–1.68) and major adverse cardiovascular events (MACE) (HR 1.55; 95% CI 1.50–1.60) [98]. In addition, the study found increased risk and an excess 12-month burden (a measure that compares the estimated incidence rate in the COVID-19 positive group to controls) for cerebrovascular diseases, inflammatory heart diseases, arrhythmias, and thromboembolic disorders compared to controls [98]. Xie et al. also investigated the impact of the severity of COVID-19 illness on the risk of post-COVID-19 cardiovascular sequelae using hospitalized/non-hospitalized status as a proxy for severity. Hazard ratios of cardiovascular outcomes in individuals hospitalized for acute COVID-19 were higher than those found in the non-hospitalized group: cerebrovascular outcomes (HR 1.53; 95% CI 1.45–1.61), dysrhythmias (HR 1.69; 95% CI 1.64–1.75), inflammatory diseases of the heart or pericardium (HR 2.02; 95% CI 1.77 to 2.30), and ischemic heart disease (HR 1.72, 95% CI: 1.65–1.79). These risks remained elevated even after stratifying by age, sex, race, obesity, smoking, hypertension, diabetes, chronic kidney disease, hyperlipidemia, or cardiovascular disease [98]. These results demonstrate the persistence of cardiovascular sequelae post-COVID-19, and find that initial disease severity is a robust determinant of long-term cardiovascular impairments.

Bowe et al. used the same cohort of U.S. veterans and extended the follow-up to two years, finding that the risk of post-COVID-19 sequelae is highest in the immediate post-acute phase (30–90 days post-COVID-19), and that although risk attenuates over time, it is still prevalent two years after infection, especially among hospitalized individuals [44]. As seen in Table 1, risk of death is most pronounced in the 30 to 90 days post-COVID-19, with the non-hospitalized cohort over two times more likely to die and the hospitalized cohort over six times more likely to die compared to uninfected controls. Risk becomes non-significant at one year for the non-hospitalized group and attenuates, but remains significantly elevated, for the hospitalized group. Unsurprisingly, risk across sequelae and across time is higher in the hospitalized cohort compared to the non-hospitalized cohort; however, many cardiovascular sequelae for the non-hospitalized cohort remain significantly elevated at one year, including risk of heart failure, coagulopathy, atrial fibrillation, bradycardia, angina, and non-ischemic cardiomyopathy. At two years, in the non-hospitalized group, significantly higher risk persists only for coagulopathy and bradycardia. In the hospitalized cohort, however, 63% of cardiovascular sequelae are still significantly elevated at two years post-COVID-19. These results are particularly compelling due to the large sample drawn from a comprehensive national healthcare database with lengthy follow-up and indicators of disease severity.

Findings from Xie et al. and Bowe et al. are consistent with other studies that find hospitalized COVID-19 patients are at a significantly higher risk for any cardiovascular event weeks to months after infection compared to non-hospitalized controls [44,91,98,99]. Furthermore, compared to non-hospitalized controls, the risk of subsequent hospitalization for a cardiovascular event was higher in those admitted to the ICU (HR 3.47, 95% CI: 3.20 to 3.76) than the non-ICU hospitalized (HR 1.96, 95% CI: 1.85 to 2.09) [99].

In addition to cardiovascular outcomes from large-scale studies that rely mostly on medical records, several clinical studies have investigated the effects of COVID-19 on vascular dysfunction. Research suggests that endothelial dysfunction is prevalent weeks to months after infection. Moreover, heightened COVID-19 severity is associated with endothelial dysfunction (OR 1.35, 95% CI: 1.06 to 1.71, *p* = 0.01) [100,101], and this endothelial dysfunction can occur independently of other risk factors. These results are consistent with other studies reporting reduced flow-mediated dilation (FMD), a measure of endothelial function, in young adults 3–4 weeks following infection compared to controls (2.71 ± 1.21% vs. 8.81 ± 2.96%) [102]. Ikonomidis et al. reported endothelial dysfunction at both 4 and 12 months after infection, along with only partially reverted markers of oxidative stress [103]. Studies examining the effects of COVID-19 on persistent arterial stiffness are more heterogeneous in their findings. Different groups have shown that arterial stiffness, measured either directly or indirectly, remains elevated 3–4 weeks [102], 2–3 months [104], 4 months [103], and 48 weeks [105] after infection. However, others have shown significant reductions in arterial stiffness 6 months post-infection, yet with the heart-rate adjusted augmentation index and circulating ICAM-1 remaining unchanged from 1 to 6 months [106]. These data suggest the large artery stiffening related to COVID-19 can be maintained for months after infection.

The increased risks of cardiovascular events and cardiovascular sequelae are likely due to multiple, and sometimes interrelated, factors, including direct viral toxicity, inflammation, thrombosis, autoimmunity, and accelerated atherogenesis, with the importance of the different pathophysiological mechanisms varying over time [90,91,107]. The inflammatory response, which has been implicated in numerous studies, has multiple components, including the “cytokine storm” and cell-mediated and hormonal responses. Furthermore, inflammation is closely linked with coagulopathies. Indeed, Cervia-Hasler et al. [108] recently reported complement dysregulation, particularly perturbed terminal complement complex (TCC) formation via increased soluble C5bC6- and decreased C7 complexes in COVID-19 patients at 6 months of follow-up. This aberrant complement response likely leads to tissue damage via increased TCC insertion, and was associated with thrombo-inflammation via increased platelet-monocyte aggregates, endothelial activation (i.e., raised von Willebrand factor and red blood cell lysis), and low antithrombin III levels. Additionally, the risk of autonomic dysfunction is elevated post-COVID-19, and this may play an important role in some cardiovascular sequelae, including sinus tachycardia, bradycardia, vasovagal syncope, chronotropic incompetence, postural orthostatic tachycardia (POTS), hypertension, and hypotension [109,110].

Adequate cardiovascular health is necessary to safely perform firefighting work. Furthermore, our group has highlighted how firefighting can acutely perturb cardiovascular physiology and blood coagulation [111]. Of particular concern is the elevated risk of thrombotic events in the months and years following firefighting and the heightened coagulatory potential that has been reported in response to firefighting activity. This interaction could enhance the risk of firefighters suffering from a major cardiac event during an active fire, which is already the leading cause of death in the service.

## 7. Cardiorespiratory Fitness

Cardiorespiratory fitness (CRF) is an integrative measure that reflects the ability of the pulmonary, cardiovascular, and muscular systems to work together to effectively supply oxygen to the muscles during maximal work/exertion. As such, it is a powerful indicator of the proper functioning of the systems discussed above under exercising conditions. Given that infection with SARS-CoV-2 can impact the musculoskeletal, pulmonary, and cardiovascular systems, it is unsurprising that studies consistently find decrements in CRF following infection with SARS-CoV-2 [112,113,114,115,116,117]. Reductions in CRF seem most pronounced in the weeks immediately following illness, attenuating over time [113,115]. For those with severe disease, decrements in CRF appear more substantial compared to those who experienced a milder illness [112,115,116]. However, there is also evidence that even mild to moderate illness can impact CRF many months post-COVID-19. One study investigated 127 healthcare workers (predominately female) who routinely had CRF assessed via cardiopulmonary exercise testing (CPET) so that there was both a baseline and post-COVID-19 comparison. At a mean of almost one year after SARS-CoV-2 infection, those who had mild to moderate COVID-19 (n = 40) experienced a significant decrease (3.12 mL/kg/min) in CRF, while no significant decrease was found among the 87 workers who did not have COVID-19 between CPET evaluations [117].

A study investigating the impact of infection with SARS-CoV-2 on the CRF of firefighters was recently conducted by D’Isabel et al. [118]. The study cohort included 103 firefighters, mean age 40 ± 9 years, who experienced mild to moderate COVID-19 and had yearly occupational CPET evaluations done in 2019 (pre-COVID-19) and again in 2020 (post-COVID-19). On average, CRF declined by 2.55 mL/kg/min (7.3%; *p* < 0.001) a mean of 110 ± 78 days after SARS-CoV-2 infection. This decrease far exceeds the estimated 1% per year (10% per decade) decrement that might be expected due to aging [119,120]. The study also found that oxygen consumption at lactate threshold was decreased by 24% at the post-COVID-19 measurement, suggesting that peripheral oxygen utilization was a major contributor to the decreased CRF. By almost one year after having COVID-19, the predicted change in CRF values based on regression analysis approaches zero; however, the observed data reveal considerable variability in the CRF decrements and indicate that for some firefighters, decrements in CRF persisted nearly a year after infection. Given the importance of CRF for the successful job performance of firefighters, and the increase in cardiovascular risk associated with lower CRF, there is an urgent need to better understand changes in CRF and mitigation strategies to address CRF decrements post-COVID-19.

## 8. Limitations

This review synthesized the available literature on the sequelae of COVID-19 on three body systems and considered the impact of the sequelae on the occupational readiness of firefighters. Although there has been a tremendous amount of research activity since the onset of COVID-19, there remains a great deal that is unknown and a great deal that is still being investigated. Some of the challenges with interpreting and/or synthesizing the literature arise because of the nonuniform COVID-19 research landscape. Researchers have used different nomenclature and definitions and investigated different groups of patients, with a variety of characteristics and severities of initial illness. The widely divergent course of illness, recovery, potential for multiple infections, and differing vaccination status among individuals also present a challenge when synthesizing the literature. Furthermore, much of the data that has been published was collected before breakthroughs and widespread use of vaccines and COVID-19 therapeutics, which could affect the results of future research.

## 9. Conclusions

Millions of people continue to struggle with the long-term consequences of COVID-19. Infection with SARS-CoV-2 results in an array of post-acute sequelae that affect the musculoskeletal, pulmonary and cardiovascular systems. Sequelae appear most prevalent in the immediate post-acute phase, and although risk often appears to attenuate with time, some sequelae remain prevalent even at two years post-COVID-19. Initial disease severity heightens the risk of experiencing many post-acute sequelae across the three systems studied.

While post-acute COVID-19 conditions can present a serious challenge to all individuals, the sequelae explored in this review that are specific to the musculoskeletal, pulmonary, and cardiovascular systems are of particular concern for public safety personnel, like firefighters, who must perform strenuous duties as part of their work. Similarly, the health care system has been seriously challenged by dealing with the number of individuals suffering from the variety of post-acute sequelae from COVID-19, especially given the lack of clear guidance on how to successfully treat the numerous conditions presenting. Moreover, providing care to those in strenuous, tactical occupations may be uniquely challenging. Given the number of people affected, and the severity and complexities of the post-acute sequelae that arise after COVID-19, additional research is desperately needed.

## Figures and Tables

**Table 1:** Relative risk of post-COVID-19 cardiovascular sequelae up to 2 years after infection by hospitalization status.

			Days Post-Infection				
	Sequela		90	180	360	540	720
	Death	NH	***2.22***	***1.17***	1.00	0.96	0.99
		H	***6.25***	***1.75***	***1.41***	***1.42***	***1.29***
	Hospitalization	NH	***1.45***	***1.18***	***1.06***	***1.06***	1.04
		H	***6.83***	***3.14***	***2.66***	***2.64***	***2.57***
**Ischemic Heart Disease**	Acute Coronary Disease	NH	***1.73***	1.07	0.93	1.09	0.93
		H	***18.32***	***4.31***	***1.83***	***1.91***	***1.45***
	Angina	NH	***1.47***	***1.20***	***1.25***	***1.31***	1.15
		H	***5.20***	***3.09***	***2.03***	***1.79***	***2.32***
	Myocardial Infarction	NH	***1.62***	1.05	1.01	1.11	0.97
		H	***15.89***	***4.32***	***1.78***	***2.13***	***1.56***
	Ischemic Cardiomyopathy	NH	1.31	1.23	1.05	1.03	1.10
		H	***6.19***	***2.43***	***2.05***	***1.97***	1.18
**Cardiac Failure**	Cardiac Arrest	NH	1.46	0.79	0.85	0.73	1.01
		H	***34.78***	***14.30***	1.17	1.94	1.39
	Cardiogenic Shock	NH	0.77	1.45	0.72	0.98	0.97
		H	***19.41***	***6.51***	***2.15***	1.89	1.84
	Heart Failure	NH	***1.91***	***1.41***	***1.22***	1.04	1.06
		H	***13.13***	***3.00***	***2.00***	***1.95***	***1.47***
	Nonischemic Cardiomyopathy	NH	***1.72***	***1.44***	***1.13***	1.06	1.00
		H	***7.89***	***4.08***	***2.10***	***2.63***	1.15
**Dysrhythmias**	Atrial Fibrillation	NH	***2.14***	***1.24***	***1.20***	***1.12***	0.95
		H	***16.34***	***3.22***	***1.79***	***1.49***	***1.63***
	Atrial Flutter	NH	***1.58***	1.32	1.16	0.95	0.96
		H	***10.67***	***3.42***	1.48	1.27	0.79
	Bradycardia	NH	***1.45***	***1.27***	***1.19***	***1.29***	***1.18***
		H	***8.72***	***2.24***	***1.75***	***1.81***	***1.51***
	Tachycardia	NH	***2.01***	***1.26***	1.06	1.03	1.14
		H	***19.83***	***4.08***	***2.34***	***2.36***	***2.66***
	Ventricular Arrhythmia	NH	***1.89***	***1.42***	1.01	1.11	1.04
		H	***16.99***	***4.67***	***1.58***	***1.95***	***2.22***
**Coagulation**	Pericarditis	NH	***1.87***	1.23	1.13	1.05	1.15
		H	***17.54***	***5.47***	***2.38***	1.55	1.19
	Anemia	NH	***1.82***	***1.15***	1.01	1.02	0.94
		H	***11.16***	***2.74***	***1.58***	***1.57***	***1.50***
	Coagulopathy	NH	***2.00***	***1.44***	***1.15***	***1.13***	***1.23***
		H	***12.86***	***3.57***	***2.62***	***2.62***	***1.84***
	Deep Vein Thrombosis	NH	***3.30***	***1.71***	1.14	0.96	1.08
		H	***17.63***	***2.88***	***1.95***	***2.52***	***2.13***
	Pulmonary Embolism	NH	***4.99***	***1.94***	0.87	1.11	0.95
		H	***45.55***	***6.66***	***2.16***	***1.54***	***1.65***
	Venous Thromboembolism	NH	***2.90***	***1.41***	1.07	1.07	0.86
		H	***20.36***	***5.69***	***1.97***	***1.78***	1.23

Table adapted from Figure 1 in Bowe et al. [44]. Significant findings bolded and italicized. The last day in the time-period after infection is labeled (90, 31–90 days; 180, 91–180 days; 360, 181–360 days; 540, 361–540 days; 720, 541–720 days). H, hospitalized for acute illness; NH, not hospitalized for acute illness.

## Data Availability

Not applicable.

## Abbreviations

IDLH	Immediately dangerous to life and health
SCBA	Self-contained breathing apparatus
DALYs	Disability adjusted life years
SDH	Succinate dehydrogenase
EMS	Emergency Medical Service
FEV1	Forced expiratory volume in 1 s
FVC	Forced vital capacity
DLCO	Diffusing Lung Capacity for Carbon Monoxide
HRCT	High-resolution computed tomography
MACE	Major adverse cardiovascular events
FMD	Flow mediated dilation
TCC	Terminal complement complex
POTS	Postural orthostatic tachycardia
CRF	Cardiorespiratory fitness
CPET	Cardiopulmonary exercise testing
